# High turnover in clinical dietetics: a qualitative analysis

**DOI:** 10.1186/s12913-020-06008-5

**Published:** 2021-01-06

**Authors:** Sarah Hewko, Amirah Oyesegun, Samantha Clow, Charlene VanLeeuwen

**Affiliations:** University of Prince Edward Island, 550 University Avenue, Charlottetown, PE C1A 4P3 Canada

**Keywords:** Canada, Health workforce, Hospital administration, Nutritionists, Personnel administration, hospital, Personnel turnover

## Abstract

**Background:**

Relationships between dietitians and other healthcare providers can impact the degree to which patient care is collaborative; inefficient communication can lead to suboptimal care. It takes time for multidisciplinary team members to build collaborative, trusting relationships. For this reason, frequent dietitian turnover is of concern. Consequences include fewer referrals to clinical dietetic services and limited provider continuity. The characteristics of clinical dietetic jobs associated with high turnover have not been identified. We predicted that managers would identify disease prestige as having an impact. In this study, we aimed to explore: 1) characteristics of clinical dietetic jobs associated with the highest turnover, and 2) consequences of high turnover on patients and managers of clinical dietitians.

**Methods:**

Research assistants conducted semi-structured interviews with ten managers of clinical dietitians in the Canadian public healthcare system. We employed a constant comparative approach to thematic analysis. We classified themes related to turnover as either avoidable or unavoidable.

**Results:**

Sub-themes under avoidable turnover included lack of manager support, growth opportunities, burnout/workload, tension/conflict and hours of work. Sub-themes under unavoidable turnover included life-stage/life-events and geography. We also identified themes related to consequences of turnover, including: burnout/workload, client/patient impact, tension/conflict, cost and gap-specific. As predicted, prestige was perceived as playing a role in triggering dietitian turnover. Managers observed high turnover resulting in low provider continuity and limiting patient access to dietitians.

**Conclusions:**

Managers of publicly-employed dietitians identified many factors as contributing to high turnover. Future prospective research, incorporating the objective measure of turnover and multi-method analysis of work characteristics and work setting, would be of value in the identification of characteristics of clinical dietetic jobs associated with high turnover and the consequences of high turnover on patients and managers of these staff.

**Supplementary Information:**

The online version contains supplementary material available at 10.1186/s12913-020-06008-5.

## Background

Registered Dietitians (RDs) are healthcare professionals that have specialized in human nutrition. Unlike nurses employed in hospitals, hospital-based RDs commonly provide clinical coverage for more than one patient unit [1]. Relationships between RDs and other healthcare providers can impact the extent to which patient care is collaborative and the relative efficiency of interprofessional communication; inefficient and ineffective communication can lead to suboptimal patient care [2]. For this reason, healthcare leaders should be concerned when there is frequent turnover in an RD position serving a unit or unit(s).

With each new RD assigned to a role, there is a time lag while the individual becomes acclimatized to the culture and norms of the unit (both clinical and organizational) and builds relationships with other members of the unit(s)’ multidisciplinary team. Few have explored the impact of workforce instability on the performance of healthcare providers [3] and the peer-reviewed literature reveals little about how characteristics of work settings affect RDs [4]. Turnover is typically expressed as the proportion of staff who have moved out of a particular job within the last year [3]. Turnover is also frequently measured at the level of the facility or organization (e.g., turnover rates among healthcare aides [5], within a specific profession (e.g., nursing [6]) or, less commonly, at the industry level (e.g., manufacturing [7]). For our purposes, turnover is defined as movement of an RD out of a job followed by a search for a temporary or permanent replacement (referred to by Burgess et al. (2000) as worker flow [7]). We have no objective working definition for high turnover, as turnover in dietetics has been studied very little and rates of turnover vary across industries [7]. Rather, we are considering turnover in relative terms — high turnover positions are those with the most frequent turnover in a clinical RD manager’s portfolio.

Registered Dietitians in both the United States of America (US) and Canada complete, at a minimum, a baccalaureate degree (most commonly in dietetics) and complete an accredited internship before being eligible to write the licensure exam [8,9]. The International Confederation of Dietitian Associations, an organization made up of members of national dietetic associations, has worked to develop international standards for education, competence, ethics and good practice [10], which has facilitated reciprocity agreements for RDs wanting to practice in other countries (e.g., US dietitians wanting to work in Canada, the Netherlands, the Philippines or Ireland). The title of dietitian (in Canada [11]) and dietician (in the US [8]) is protected, such that only those with the appropriate credentials can use the title.

Courses in dietetics were first offered in 1914, but the profession’s prominence rose most significantly in the 1940s as dietitians were employed as civilians in military hospitals, primarily to manage foodservice operations. It was in the late 1940s that dietitians first became involved in providing dietary treatments in clinical settings [12]. According to the Canadian Institute for Health Information, there were 12,170 RDs in Canada in 2018 [13]. Although there is no official tally of RDs working in clinical positions, a Dietitians of Canada report on the workforce in British Columbia indicates that 48% of RDs in that province were employed in hospitals [14]. The proportion employed in hospital would be similar across Canada.

Clinical dietitians often work as part of a multidisciplinary team and would commonly be the only RD on each team. Decisions regarding the quantity of RD required (in full-time equivalents) are typically based on actual or expected patient volume (e.g., number of beds on the unit) [15]. Clinical dietitians are classified as allied health professionals alongside physiotherapists, occupational therapists, speech language pathologists and others. As part of their role, clinical RDs complete nutrition assessments; plan, implement and evaluate nutrition interventions; coordinate nutrition care with other healthcare providers; attend formal rounds and care conferences, and; ensure thorough documentation of their work in the medical record [16].

The degree to which clinical nutrition expertise is utilized depends on physicians and other health professionals having a positive attitude toward these services [17]. In the Canadian public healthcare system, physician referral is the primary avenue for access to RD services (whether inpatient or outpatient) [18]. Similarly, physician referral is primary in Australia [19], Austria [20] and the US (Medicare requires physician referral [21]). Who is eligible to make referrals to the RD, other than physicians, varies across settings and organizations. In the absence of clear protocols to guide referral or consultation, it is difficult to predict which circumstances will trigger RD consultation [17,22]. Physicians’ perceptions of RD services will depend, at least in part, on their relationship with the RD(s) providing service to their patients. Patient outcomes are improved when health professionals communicate and collaborate efficiently [2,23]. In their first year in a position, only 80% of health professionals felt comfortable providing suggestions to referring physicians; this rose to 100% among those with tenure > 4 years [24]. The time required for RDs to build collaborative, trusting relationships with physicians on the unit(s) they serve is extended when the unit is served by multiple physicians.

Consequences of frequent turnover are not limited to those associated with a reduction in *initial* consultations — such as the prolonged lengths of stay [18,25,26], increased infection rates [18] or delayed healing [18] that can result from malnourished hospital patients not being assessed or referred to the RD early in their stay. Longer-term patients may also receive suboptimal care as a result of high turnover. Consistency in care provider, or *provider continuity*, is one component of continuity of care [27]. Inconsistent care can decrease a provider’s ability to interpret changes in the patient’s behavior or appearance and can weaken rapport [27,28]. Patients may become frustrated at having to repeatedly share their medical history and existing concerns [28]. Notably, provider continuity may matter more to patients who are at the end of life, are elderly, have chronic conditions and/or who have complex histories [29].

Following a systematic review of the literature exploring associations between continuity of care and patient outcomes, van Walraven et al. (2010) concluded that increased provider continuity predicted greater patient satisfaction and improved patient outcomes [30]. More specific to allied health professionals, results from a US study on the impact of provider continuity in outpatient physiotherapy indicated that patients with lower provider continuity were less likely to experience functional improvements and were more than twice as likely to require hospitalization than those who experienced a high level of provider continuity [27].

In this study, we aimed to explore: 1) characteristics of clinical dietetic jobs in Canada associated with frequent turnover and 2) consequences of high turnover for patients and for managers of clinical RDs. Despite the lack of literature available to guide prediction of findings, we hypothesized that the highest turnover would be in general medicine and long-term care (gerontology) RD positions. This hypothesis was based on the principal investigator’s professional experience as a manager of clinical RDs and on literature related to variation in the prestige of particular diseases and medical specializations.

Association with a higher prestige specialty or disease can furnish both material and nonmaterial advantages [31] such as greater autonomy, higher pay and elevated social standing [32]. Higher prestige specialties and disease conditions are often allocated a disproportionately large portion of available resources. Consequently, patients with lower prestige conditions may be offered lower quality treatments, further exacerbating health inequities [31]. Although only a small proportion of RDs have a formal specialist designation (e.g., Certified Diabetes Educator [33], Certified Nutrition Support Clinician [34]) prestige can be seen as inherent to positions serving patients diagnosed with high prestige disease conditions.

In general, categories of disease more common in young patients [31,35]; those allowing for greater demonstration of “power” (e.g., specialist vs. general practitioner) [31]; those whose treatments do not yield helplessness or disfigurement [35], and; those that can be resolved or cured via innovative or advanced technological or surgical interventions [31] are apportioned greater respect. Lower prestige is conferred on delocalized diseases – that is, those not confined to a particular location in the body [31]. Researchers have noted that the relative prestige of diseases has remained stable over time [35], with evidence that general practice and gerontology consistently sit at the bottom of the prestige hierarchy [36].

## Methods

In this study, both quantitative and qualitative methods were employed to identify key characteristics of jobs in clinical dietetics associated with high turnover and to assess the consequences of high turnover, both for patients and managers of clinical RDs. Our quantitative results are reported in a separate publication [37]. This study was approved by The University of Prince Edward Island Research Ethics Board. The study took place in May, June and July of 2019. In this article, we will be reporting only the qualitative results.

### Sample selection and recruitment

Any individual responsible for the management of clinical RDs (supervising a minimum of three RDs) in Canada was eligible to participate. The primary investigator (SH) has an extensive personal network of clinical managers across Canada as a result of her clinical dietetics management experience. Additional contacts were identified through the Dietitians of Canada Clinical Managers Network or were identified via a targeted search in LinkedIn™. Additionally, requests for participation, in both French and English, were circulated on SH’s Twitter feed (@Sarah_Hewko).

**Fig. 1:**
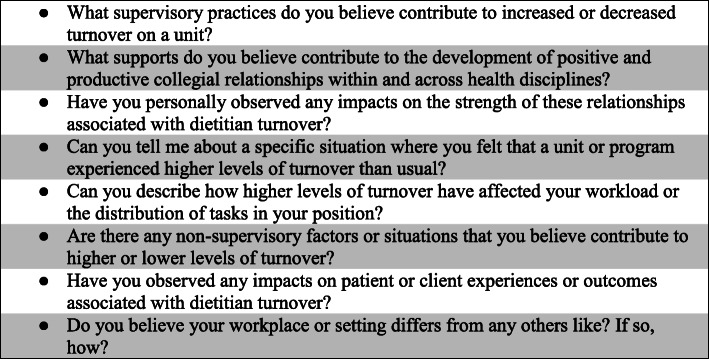
Semi-structured interview questions

Those identified as eligible respondents (*n* = 31) were contacted by e-mail by a member of the research team; a consistent e-mail script and a formal study introduction letter (see Appendix 1) were attached. Those who did not respond to the first request for participation were sent two reminder e-mails at 3 weeks intervals. Respondents were encouraged to pass information about the study along to other eligible respondents. All respondents completed a survey with primarily quantitative questions; the survey was developed explicitly for this study. The survey contents and quantitative results will be published separately. The last question of the survey was an invitation to participate in a semi-structured telephone interview on the topic of high turnover in clinical dietetics. We aimed to have 10–15 respondents. All who expressed interest in being interviewed were approached to participate. Questions were crafted by CV based on existing literature (e.g. [24, 31, 35],) and on the clinical dietetic management experience of SH (see Figure 1 for a list). In order to preserve the anonymity of managers working in rural or remote communities, we did not collect information such as gender, age, place of employment, tenure in job or job title from respondents. All interviews were conducted by female undergraduate research assistants (AO and SC), both majoring in foods and nutrition. Both research assistants had reviewed key chapters (2 and 9) in Kvale’s (1996) *Interviews* [38] and practiced mock interviews prior to conducting interviews with respondents. All interviews began with a scripted request for informed consent. Interview respondents were offered a gift card to a Canadian chocolatier valued at $25 to compensate them for the time they had committed to the project (~ 15–30 min). Each interview was conducted by telephone from the university and was digitally recorded and transcribed verbatim; recordings and transcripts were uploaded to a secure server for long-term storage. Each interview was transcribed by either AO or SC and then audited for accuracy by the other. We elected not to calculate inter-rater reliability as it has been repeatedly demonstrated to be ineffective in demonstrating the reliability of qualitative research [39]. Respondents were sent transcripts of their interviews for review (member-checking); they were notified that the transcripts would be incorporated into analysis “as is” if there was no response within 2 weeks. We have lightly edited the presented quotes to remove discourse markers and filled pauses.

### Analysis

The analytic approach for this study was both deductive and inductive [40]. We conducted thematic analysis of qualitative data, an approach that facilitates researchers gaining a better understanding of respondents’ influences and motivations and how these impact their responses to events [41]. Guided by Glaser and Strauss’ (1967) constant comparative approach, all research team members gathered after data collection but before analysis to develop an initial codebook [42,43]. Research assistants (AO and SC) independently applied the themes identified in the codebook to analysis of all interview transcripts and were encouraged to create new themes/codes as needed. Following this round of independent, focused coding, AO and SC met to compare. Disagreements were discussed until consensus was reached. SH and CV were accessible in cases where consensus could not be reached [42,43].

## Results

Ten respondents from among those who had completed the survey (*n* = 20) were interviewed. We classified themes related to reason for turnover as either avoidable or unavoidable (see Table 1). Sub-themes under avoidable turnover included lack of manager support, growth opportunities; burnout/workload; tension/conflict, and; hours of work. Sub-themes under unavoidable turnover included life-stage/life-events and geography. We also identified themes related to consequences of turnover including: burnout/workload, client/patient impact, tension/conflict, cost and gap-specific.

**Table 1:** Themes Identified

Turnover		
***Avoidable***	***Unavoidable***	Consequences of Turnover
**● Managerial support**: *whether due to absence, incompetence or manager overload* **● Growth opportunities**: *lateral, vertical, towards “prestige”* **● Burnout/workload** **● Tension/conflict**: *including inter- and intra-professional and undervaluation of the RD role* **● Hours of work**: *full- or part-time*	**● Life-stage/events** **● Geography**	**● Burnout/workload**: *supervisor- and/or team* **● Client/patient impact** **● Team dysfunction** **● Cost** **● Gap-specific**

### Avoidable turnover

All ten managers addressed manager support as a factor contributing to (or preventing) high turnover; they identified both supportive and unsupportive practices and approaches. Manager characteristics and/or practices identified as contributing to higher turnover included inflexibility (P02,04,06–08); micromanagement (P02,07,08), such as “*when the manager is too involved and wants to control all of their* [an RDs] *activities*” (P07); unresponsiveness (P04,06,07), and; inattentiveness (P04,06,07). Dictatorial management styles were identified as hindering employee retention (P04,06), while providing autonomy (P05,07,08) — including the freedom to make mistakes— was associated with reduced turnover.

A trusting relationship between employee and manager was the most frequently mentioned factor positively influencing retention of RDs (P02,05–07,09) – “*some of the things dietitians have told me is that … they feel like they know that we’ll go to bat for them, that we will stand for them when it’s difficult*” (P07). Managers felt that their RD employees particularly valued their responsiveness in answering questions and in helping them to solve problems they experienced (P03,05,07). This included timely action to take RDs’ problem(s) up as high in the organization as needed to achieve a solution. Managers recognized that staff also appreciated a “listening” ear, with no expectation of specific action by either manager or RD (P01,07,09).

Practices associated with employee retention by managers included: providing informal opportunities to “touch base” (P01, P07); exhibiting transparency by clearly outlining expectations and providing rationale for either the status quo or new initiatives (P04,07,09); making time for collaborative problem-solving sessions (P04,05,09); recognizing RD accomplishments and value (individually and collectively) (P04,05); demonstrating empathy during interactions with staff (P06,07); treating staff equitably (P05,09,10), and; providing active support for RDs to achieve personal and professional goals (P04,10). Managers (P06, 07) felt that an understanding of the role of the dietitian, frequently based on personal experience as clinicians in similar roles, was of value in retaining staff. One manager summarized many key points as follows:

“*I think a leader needs to let people do their work … on their own and learn on their own from some of their mistakes … it needs to be a safe environment to try new things, to challenges one’s self and then people grow. If not, they leave.”* (P05).

The presence or lack of opportunities for growth, whether expressed as a desire for expanded skills and expertise, for prestige, or for advancement was reported as a factor impacting turnover by nine of ten managers. Specifically, as P01 noted, RDs desired “*to have education that sustains practice*.” Managers (P02,03,05) acknowledged that RDs often sought “*more challenging roles*” (P02), sometimes in a particular specialization or area of passion for them (P08). Such specialization may not have been attainable or possible within the employing organization. One manager stated explicitly:

“*sometimes I think we all move to other health authorities just because there’s some interesting positions –there’s a potential to specialize in different areas that you couldn’t do here*” (P03).

Managers most commonly reported prestige or preference as linked to a particular facility (P05,08,09), rather than as associated with an individual program or field of practice. Manager P05 commented on the popularity among RDs of working at the largest, acute care hospital and how that had led to smaller, community hospitals losing “*some of their dietitians because they want to come here.*” Holding a specialized position at this site was associated with additional opportunities for growth, development and recognition.

In the case of pediatrics, P09 noted that there was a certain cachet to treating children who “*will do anything they can to get better*” with “*parents who will do anything it takes to make their kids better.*” She noted that in adult care:

“*sometimes the conditions are complex and chronic and sometimes actually coming from choices in the past*” – “*being told you* [the patient or client] *have to change things is not always welcome, especially when it’s having to do with food or alcohol or anything like that*” (P09).

In relation to advancement, manager P03 noted a lack of positions to form a career ladder within their health authority. In smaller health authorities, there may be very limited turnover in the few existing advanced RD roles, which makes it infeasible for others to ever advance within the organization.

Eight of ten respondents identified burnout and heavy workload as contributors to turnover in their interviews. One manager (P01) reported “*workload that’s not congruent with the amount of hours that are expected*.” As a result, staff can experience “*extreme stress or distress coming to work*” as there “*is not enough time to finish their work*” (P04). Two managers (P02,03) noted that uneven workload distribution across positions contributed to turnover.

Tension and conflict were noted as factors integral to turnover by seven of ten respondents. Three referred to conflict within the profession, five to interprofessional conflict and two to conflicts in relation to undervaluation of the RD role. Conflicts among RDs can result from having to share “*very small cramped*” (P06) workspace. Such conflicts can be particularly damaging as “*they* [RDs] *are supposed to share their workload, cover for each other*” and “*work cooperatively*.” For some returning to the dietetic department could help them to “*escape*” from a hypercritical environment – “*like the gossiping and whatever goes on on the unit*” (P03). This manager suspected that:

this “*kind of escape … helps people to be able to stay longer in their positions and have less turnover because … they have support from their coworkers to not get sucked into that kind of attitude*” (P03).

Interprofessional conflict was often closely tied to undervaluing the role of the RD. Problems can arise when “*other care providers*” try “*to do the work of the dietitian*” or are not “*willing to accept the dietitian as the nutrition professional*” (P07). One manager (P07) commented on the current climate, where “*everyone thinks they can do the nutrition component*” and how this can leave RDs feeling frustrated. Another manager noted that in the past, on their eating disorders unit, a lack of trust had developed when the RD:

“*would make a recommendation, leave the unit, go see another patient … come back the next day and their recommendation would not be followed. Something else would have been suggested either by the nurse or pharmacy, physician, whatever, so they—it got to a point where they felt discouraged … why am I trying so hard when I have such a heavy caseload to do my assessments and all those recommendations when as soon as I turn, somebody goes in and changes it and they don’t even let me know they disagree, they just go ahead and change it*.” (P09).

Four of ten managers attributed some turnover to the full-time equivalence of available positions, with some RDs expressing a desire for part-time and others for full-time. Manager P01 noted that the “*part-time positions don’t seem to turn over quite as much.*” In their experience, once employees had children they tended “*not to want to come back full-time*” and, if they did come back full-time, they moved “*into a part-time position as soon as they*” could. Despite these positions being “*coveted from a staff perspective*,” the organization did not “*want part-time positions*” or job shares (P01). In fact, this manager’s organization had merged many part-time positions to form full-time positions (P01).

In contrast, a manager of RDs in rural areas (P02) reported seeing higher turnover in part-time positions, attributing this, at least in part, to the employee-paid expense and time associated with travel to and from work sites. A preference for full-time was echoed by P06 and P10, with both noting that RDs began in part-time positions and remained only until a full-time position became available.

### Unavoidable turnover

Life-stage was noted by seven of ten managers as a reason for turnover. It is well known that dietetics is a female dominated profession and many RDs are in the “*the time of their lives that they want to start a family*” (P04). Employees will also leave their positions “*because their family is moving*” (P05) or “*their husband gets transferred*” (P07). Retirement was also considered an unavoidable reason for turnover. In some instances the health authority can be blindsided by how much a retiring RD actually did: e.g., “*when they were trying to package her responsibilities they didn’t even know all the jobs she did and some didn’t get done and there was risk*” (P06).

Geography, or the location of the RD position, was another factor contributing to turnover. In some health authorities, many new hires were making a move to a more rural part of Canada where they may feel isolated. If “*people are here without any family or any of their friends, sometimes that is kind of the issue of why they moved away*” (P03). In other cases, it may be that the geographical coverage area is too large:

“*When you give a dietitian too much space to try and cover it is hard for them to make meaningful connections in all of those communities …*” and “*until you have someone who actually wants to be in that community, who is from that community, who’s got a partner that lives in that community [laughs] … you have lots of turnover in that community*.” (P07).

### Consequences of turnover

Burnout and high workload were identified not only as triggers, but also as consequences of turnover. All ten managers indicated that burnout and high workload related to turnover impacted their own workload and job quality, while nine identified similar effects on remaining team members (including RDs). A common sentiment was that managers were “*constantly recruiting new staff*” (P01). Recruitment and hiring were “*quite a process … from getting approvals and getting job postings to interviewing*” (P02), training and orientation.

These tasks can take “*time away from actually leading practice, addressing practice issues and looking at … expanding programs, securing funding … making proposals to advocate for the profession*” (P04). This can perpetuate circumstances where coverage of particular units or programs is insufficient to meet the dietetic needs of clients and patients.

Various impacts on the team were reported to result from having to pick “*up the slack for people that have gone on*” (P02) and/or from the increased workload associated with training new staff (P02,03,06,09,10). Staffing changes can create a “*domino effect*” (P09) where multiple people shift positions as a result of the first turnover event. P09 noted how the team must adapt when there is turnover and learn to “*trust the new person coming in*” (P09); frequent turnover can “*decrease trust from the unit level in our department because we aren’t able to meet the demands of the unit*” (P10).

All ten respondents identified impacts on clients and patients resulting from RD turnover. Commonly noted impacts included: delayed nutrition care (P02, 03,04,06,10), in particular delays triggered by lengthened waitlists (P03,04,06,10) and cancelled clinics (P04); prolonged hospital stays (P02,04), which may result from delayed discharge planning (P04) and/or malnutrition (P10), and; less skilled nutrition care while inexperienced RDs build experience (P07,09). These impacts were notable even when there was no gap in service as new RDs are “*usually less efficient at first so there’s still fewer people getting seen or it takes longer to get to them*” (P03). A risk of delaying RD-provided nutrition care, particularly in outpatient settings, was raised by P06:

“*when people are waiting a long time to see a dietitian … I believe that they will search out different forms of information and there is a whole pile of it that is not a very high quality in the public sphere, and I think that people may or may not engage in seeing a dietitian if they have to wait too long*” (P06).

Additionally, as noted by P10, patients or clients with time-sensitive issues/concerns, such as prenatal clients, bear greater risk as a result of delayed nutrition care.

Several respondents (P01,07,08) indicated that “*patients have seen lots of different people* [RDs] *and they feel that there’s a lack of continuity*” (P01). P08, speaking to practice in the long-term residential care setting indicated that “*residents develop relationships with the staff because*” they “*provide care to people through an extended time period*” and that there may be “*some frustration on the part of patients that they have to catch people up to what their history has been*” (P08).

Three managers commented on team dysfunction that could result from RD turnover. In rural locations, frequent turnover in what may be the only position serving the community can result in loss of “*the trust of the community*”(P07) so that the RD is no longer sought out to participate in client-care or program development because community members begin to think: If the RD is only going to be here “*a couple of months* … *why would we bring her* [or him] *into these conversations*” (P07)? This can result in the loss of *“opportunities to make a difference in the community*” (P07). In other cases, it may be that non-RD staff step in to fill the void during recruitment and orientation post-RD turnover and then have difficulty stepping back once the new RD is practice-ready (P09).

Managers (three of ten) also called attention to the cost of turnover. P01 noted how when turnover is high she has “*to train more*,” which drains her budget for RD relief. This meant that remaining RDs may no longer have had access to workload relief or back-fill when needed.

Some of the impacts of turnover were gap-specific, meaning that they occurred only when there was a vacancy in the position while awaiting a replacement RD. Gaps in service are not always the result of failed searches for new staff; “*there is often gap in service between the time a person leaves to the time a new person can come in*” (P02). In rural areas candidates can “*take the better part of a month*” before they are able to report to work (P03). Rural communities can also experience long stretches without access to an RD – in P07’s observation, communities “*get used to not using the dietitian and then … when we do get a dietitian back in that position, they* [the RD] *have to rebuild the trust and the whole practice that the previous dietitian had.*”

## Discussion

The managers of clinical RDs in our study could not identify any single factor that differentiated a high turnover position from a low turnover position. The factors they identified (see Fig. 1) are consistent with those reported in Halter et al.’s (2017) [44] systematic review of systematic reviews as being linked to job turnover and job turnover intentions among nurses. We were unable to locate research that specifically explored associations between job characteristics and the volume of turnover in a particular type of RD position (e.g., positions treating a specific disease or serving a particular subset of clients).

It is clear that turnover is not entirely avoidable, nor would it be optimal to eliminate it. In their theoretical cost-benefit framework of turnover, Abelson and Baysinger (1984) asserted that the relationship between organizational performance and employee turnover was U-shaped. That is, just as too much turnover can have a negative impact on organizational performance, so too can too little [45]. Ideally, those contributing most to the goals of the organization will remain and those contributing less than their fair share will elect to leave. Without doubt, however, is that disproportionate turnover that systematically disenfranchises a particular, vulnerable subset of the population should be avoided.

Certain forms of turnover, whether temporary or permanent, contribute to the social good. For instance, the mandate to provide maternity and parental leave is consistent with intentional legislative strategy to increase fertility rates and achieve gender equity [46]. Reported impacts of retirement on health are mixed [47–49], with some researchers indicating that retirement is associated with health improvements [50–52] and others indicating that retirement negatively impacts health [53–55]. Regardless, retirement is likely to remain a standard stage of work lives; this is especially true in healthcare, where employer-provided pension plans often incentivize early retirement [56]. Results of a recent Canadian study indicate that Registered Nurses (RNs) and allied health professionals (including RDs) retire well below the age of 65 years [57] and that caregiving responsibilities significantly increase the odds (OR = 7.6) of RNs retiring early [58]. A small RD sample size made it infeasible to test the association between caregiving and early retirement within the profession [58]. Retention schemes subsidizing support for caregivers [59, 60], facilitating flexible work arrangements [60, 61] and/or expanding leave policies to better accommodate the demands of providing care on employees [59, 60] *may* effectively deter or delay early retirements across the health workforce.

Several respondents identified prestige as a factor contributing to high turnover. Rather than specifying patient type or disease treated, managers attributed “desirability” to particular institutions — typically large institutions providing specialized acute care services. Employment in these institutions was tied to opportunities for growth and the capacity to have a far-reaching impact on RD practice. Increased stature may come to any RD employed in those institutions, regardless of the patient population they serve or diseases they treat.

Only one respondent hinted at the role that patient “*deservedness*” may play in determining the desirability of a clinical RD position. In this case, it was connected to the age of the patient or client (pediatric vs. adult). The idea that RDs may experience more negative feelings when treating patients whose condition is perceived to result from past choices is congruent with Norredam and Album’s (2007) interpretation of disease prestige as, at least partially, resulting from healthcare professionals’ impulse to attribute blame to certain groups of patients for their illness [31]. As an extension of this impulse, those with attributed blame for their condition would be considered less deserving of aid than those perceived as blameless.

Provider continuity, demonstrated in other studies to improve patient outcomes [27–30], was noted by managers to be desirable in dietetic practice and was conspicuously lacking where turnover was high. Delays in provision of nutrition therapy by an RD can have significant consequences: both financially — for the health system and the individual patient or client [62,63)], and medically — in the form of increased morbidity and mortality associated with malnutrition [63,64]. Following analysis of prospective cohort data collected in Canadian hospitals, Curtis et al. (2017) concluded that patients assessed as being severely malnourished (11%) had 53% longer lengths of stay and medical costs 55% higher than those of well-nourished patients [62]. Similar findings have been reported for malnourished hospitalized patients in Australia (26), the United Kingdom [65], Singapore [66] and the US [67] More specific to RD intervention, results of a study by Keller et al. (2015) indicate that malnourished patients seen by an RD early in their hospital stay have decreased rates of infection, improved healing and reduced lengths of stay [18].

### Strengths and limitations

There were some limitations inherent to our design. In particular, we spoke only with managers and so cannot be sure that managers’ perceived causes of high turnover in clinical dietetic positions are the same as those that would be identified by clinical RDs. That being said, the majority of managers will have worked as clinical RDs, often in the same institutions that their staff currently work in. All will have made choices to change jobs even if only to move from a previous position into their managerial position. For this reason, they have significant insight into factors impacting clinical RDs’ job-related decisions.

It is difficult to say how reflective our sample is of the broader population of Canadian clinical managers as, in order to preserve anonymity of those in rural/remote communities, respondents were not asked to provide demographic information. Also, there exists no register of clinical dietetics managers in Canada. Survey respondents were from across Canada, with nine of ten provinces represented and zero of three territories.

In employing qualitative methodology to explore the question of contributors and outcomes of high turnover in clinical dietetics, our goal was not to produce generalizable results. Rather, we hope that future research can build on and assess the consistency of these themes as they relate to high turnover in clinical dietetics.

We are also aware that there are differences in the reporting structure for RDs across Canada that may make interpreting the role of managers in preventing high turnover difficult. In some institutions and health authorities, RD management is centralized and profession-specific so that all RDs report up within a nutrition “silo” (e.g., Alberta). In others, RDs are more likely to be managed in their programs or units, with Professional Practice Leaders providing auxiliary, profession-specific support to RD employees embedded in teams across the institution or health authority (e.g., most of British Columbia).

## Conclusion

Canadian clinical managers of RDs identified many factors contributing to high turnover in their organization. As predicted, managers did perceive prestige as playing some role in triggering RD turnover. This had less to do with prestige inherent to a particular disease or patient population and more to do with the reputation and size or scope of an institution. Our respondents consistently indicated that low provider continuity arising from high turnover had impacts on patient and client access to RDs. Future research could focus on prospectively identifying positions in multiple areas and settings before conducting in-depth, multi-method analysis of the settings and work environment, while objectively measuring turnover over time. Alternately, it may be interesting to select several positions with high turnover and interview all individuals who held that position about their experience in the position and the impact of job characteristics on their decision to leave; such comparative analysis may reveal new or unexpected factors that contribute to high turnover.

## Supplementary Information

**Additional file 1.** BMC Health Services Research Appendix. This is the information letter that was provided to respondents. It is cited as Appendix 1 in the manuscript text.

## Data Availability

The datasets used and/or analysed during the current study are available from the corresponding author on reasonable request.

## Abbreviations

RD: Registered Dietitian
RN: Registered Nurse
US: United States of America
