# Supplementary material for: High turnover in clinical dietetics: a qualitative analysis
Source: BMC Health Serv Res. 2021 Jan 6;21:25. doi: 10.1186/s12913-020-06008-5 (PMC7789381; doi:10.1186/s12913-020-06008-5)
Supplement: Supplementary file 1 — Additional file 1. BMC Health Services Research Appendix. This is the information letter that was provided to respondents. It is cited as Appendix 1 in the manuscript text. [file 12913_2020_6008_MOESM1_ESM.docx]

**Appendix 1: Letter of Information**

There’s blood in the water: Characteristics of high-churn positions in dietetics

**Principal Investigator**:

Sarah Hewko, RD PhD, Assistant Professor, Department of Applied Human Sciences, University of Prince Edward Island

**LETTER OF INFORMATION FOR SURVEY RESPONDENTS**

**Invitation to Participate**

You are invited to participate in a research study that explores the characteristics of Canadian jobs in the field of clinical dietetics that are associated with the most turnover (or “churn”).

**Purpose of the Letter**

The purpose of this letter is to provide you with information so that you can make an informed decision about participating in this research.

**Purpose of the Study**

The purpose of this study is to identify the characteristics of jobs (e.g., assigned units/programs) associated with the most “churn” among Canadian clinical Registered Dietitians. This survey and interview data may be used by myself or an affiliated researcher or student to address related, secondary questions.

**Inclusion Criteria**

In order to participate in this research you must currently be in a role in which you are responsible for overseeing a minimum of three Registered Dietitians in clinical practice.

**Study Procedures**

If you agree to participate, please complete the attached survey and return it to me via e mail. I anticipate that the survey will take fewer than 20 minutes to complete. However, additional time may be required to track down information requested in the survey. Participants who express interest in participating in an interview on the topic of “churn” in clinical dietetics may be contacted for a ½ hour virtual, semi-structured interview.

**Possible Risks and Harms**

There are no known or anticipated risks associated with participating in this study. You can

decline to answer any question(s).

**Possible Benefits**

I cannot guarantee any direct benefits because of your participation in this study. However, it is my hope that the information gained through this research will help those in your position (i.e. those overseeing the work of clinical dietitians in the Canadian healthcare system) to advocate for changes to job design and/or caseload division that may lead to reduced job turnover, particularly in those areas where provider continuity is of significant value.

**Confidentiality and Privacy**

All surveys will be stored securely at the University of Prince Edward Island. All survey and interview data collected will remain confidential and accessible only to investigators of this study. If you wish to remain completely anonymous, you can mail your completed survey directly to me (see mailing address below) with no return address. In this case, consent will be implied and withdrawal from the study will not be possible. The survey document itself will not contain your name, position title or place of work. When results are published, your name will not be used or reported in any publication.

**Contacts for Study Questions or Problems**

After having participated in the study, you are free to withdraw from the study within 3 weeks of participation (in either/both the survey or interview) without repercussion or penalty. If you require any further information regarding this research project or your participation in the study you may contact Sarah Hewko at shewko@upei.ca. The plan for this study has been reviewed for its adherence to ethical guidelines by the Research Ethics Board at the University of Prince Edward Island. If you have any concerns about the ethical conduct of this study you can contact the UPEI Research Ethics Board at [reb@upei.ca](mailto:reb@upei.ca) (p-(902)620-5104).

Sincerely,

Sarah Hewko, RD PhD

Principal Investigator

Assistant Professor

Department of Applied Human Sciences

University of Prince Edward Island

Health Sciences Building, 316

550 University Ave

Charlottetown, PE C1A 4N3

shewko@upei.ca

902-566-0528

Charlene VanLeeuwen, PhD

Co-Investigator

Sessional Lecturer

Department of Applied Human Sciences

University of Prince Edward Island

cvanleeuwen@upei.ca

902-566-0691
